# Antibody screening identifies HERV-K-related immune responses as candidate biomarkers in Parkinson’s disease

**DOI:** 10.3389/fcimb.2026.1838615

**Published:** 2026-07-03

**Authors:** Elena Rita Simula, Milena Fais, Tommaso Ercoli, Somaye Jasemi, Kai Paulus, Paolo Solla, Claudio Pandino, Leonardo A. Sechi

**Affiliations:** 1 Department of Biomedical Sciences, Microbiology, University of Sassari, Sassari, Italy; 2 Azienda Ospedaliera Universitaria Sassari, Microbiologia e Virologia, Sassari, Italy; 3 Neurology Unit, University Hospital of Sassari, Sassari, Italy; 4 Neurorehabilitation Service, University Hospital of Sassari, Sassari, Italy

**Keywords:** antibodies, biomarkers, HERV-K, IRF5: interferon regulatory factor 5, Parkinson disease

## Abstract

Parkinson’s disease (PD) is increasingly recognized as a disorder involving both neurodegenerative and immune-related mechanisms. To explore potential immune alterations associated with PD, we analyzed plasma antibody responses against a panel of viral and host synthetic linear epitopes in PD patients (n = 50) and healthy controls (HCs) (n = 54). Antibody levels against Interferon Regulatory Factor 5 (IRF5), Cathepsin B (CTSB), L-asparaginase (ASRGL1), Human Endogenous Retrovirus type K (HERV-K), Interferon-α (IFN-α), Interferon- ω (IFN-ω), α-synuclein, Herpes Simplex Virus type 1 (HSV-1), olfactory receptor proteins (OLF-R), and Dickkopf-related protein 3 (DKK3) were measured. PD patients showed significantly higher antibody levels against IRF5, CTSB, ASRGL1, and HSV-1 compared with controls, while antibodies against HERV-K were significantly lower. No differences were observed for IFN-α, IFN-ω, α-synuclein, OLF-R, or DKK3. Correlation analysis revealed several associations among antibody responses. IRF5 antibodies correlated positively with CTSB, ASRGL1, HERV-K, IFN-α, IFN-ω, and HSV-1. CTSB correlated with IFN-α, IFN-ω, HSV-1, OLF-R, and DKK3, while ASRGL1 correlated with HERV-K and IFN-ω. IFN-α correlated with IFN-ω, HSV-1, and DKK3, and IFN-ω correlated with HSV-1 and DKK3. HSV-1 antibodies were also associated with OLF-R and DKK3. Notably, HERV-K antibodies showed a negative correlation with DKK3. Stratification analyses indicated that antibody levels against HERV-K were higher in patients with milder disease stages accordingly with Hoehn and Yahr scale (HY 1-4) compared with HY 5, whereas IFN-α antibodies were increased in HY5 patients. Female patients generally showed lower antibody levels than males for different targets. No differences were observed according to disease duration. Overall, these findings indicate that PD patients display selective changes in antibody responses involving antiviral and host proteins. These targets may represent potential biomarkers of immune alterations in PD, although further studies are required to clarify the underlying biological mechanisms.

## Introduction

Parkinson’s disease (PD) is a progressive neurodegenerative disorder characterized by the loss of dopaminergic neurons in the substantia nigra and by the accumulation of misfolded proteins, particularly α-synuclein (Dauer and Przedborski, 2003). Although the pathological hallmarks of PD have been well described, the mechanisms underlying neuronal degeneration remain relatively unclear. Increasing evidence indicates that PD is not solely a disorder of neuronal dysfunction but also involves complex interactions between neuroinflammation, immune activation and alterations in cellular homeostasis (Lehtonen et al., 2019; Arena et al., 2022; Tansey et al., 2022).

In recent years, growing attention has been directed toward the role of the immune system in PD. Both innate and adaptive immune responses appear to be altered in patients (Schonhoff et al., 2020), and inflammatory processes (Pajares et al., 2020) have been reported in both the central nervous system and peripheral compartments. In particular, interferon-related pathways and antiviral immune responses have been suggested to contribute to inflammatory signaling and cellular stress in neurodegenerative conditions (Chen et al., 2025; Heiden et al., 2026). Molecules involved in these pathways, including interferon regulatory factors and type I interferons, play a key role in the control of viral infections but can also influence cellular metabolism, protein turnover and immune regulation (McNab et al., 2015; Sun et al., 2015; Fritsch and Weichhart, 2016; Zannikou et al., 2024).

Viral infections have also been proposed as potential contributors to chronic immune stimulation in neurodegenerative diseases. Herpes simplex virus type 1 (HSV-1) is a neurotropic virus capable of establishing lifelong latency in the nervous system with periodic reactivation (Daheshia et al., 1998). These reactivation events can trigger innate immune responses and interferon signaling, potentially contributing to sustained immune activation (Caggiu et al., 2017; Amin et al., 2019). In parallel, endogenous viral elements integrated in the human genome, such as human endogenous retroviruses (HERVs), have been increasingly investigated in neurological disorders (Küry et al., 2018; Simula et al., 2025). HERV-K, one of the most biologically active members of this family, can be transcriptionally regulated by inflammatory and interferon-related signals and has been implicated in several neurodegenerative diseases (Simula et al., 2024; Le et al., 2025). However, its potential relationship with immune responses and cellular pathways in PD remains poorly understood.

Alongside immune and viral factors, alterations in cellular mechanisms responsible for protein degradation and metabolic homeostasis are central features of PD pathology (Caggiu et al., 2019; Bi et al., 2021; Mark and Tansey, 2025). Lysosomal enzymes such as cathepsin B (CTSB) are involved in protein turnover and autophagic degradation (Yadati et al., 2020; Xie et al., 2023), processes that are frequently impaired in PD (Lu et al., 2024; Sharma et al., 2025). Similarly, proteins involved in amino acid metabolism and protein quality control, including ASRGL1, may contribute to the regulation of cellular homeostasis under conditions of stress (Iraci et al., 2017; Garcia-Montojo et al., 2024). In addition, signaling pathways involved in cellular survival and stress responses, such as the Wnt pathway, have also been implicated in neurodegenerative processes (Serafino and Cozzolino, 2023). Dickkopf Wnt signaling pathway inhibitor 3 (DKK3), a modulator of Wnt signaling, has been associated with cellular stress responses and tissue remodeling (Zenzmaier et al., 2013; Li et al., 2017).

Given the interplay between immune activation, antiviral responses and cellular stress pathways, the analysis of circulating antibody responses against viral and host targets may provide useful comprehensions into biological processes associated with PD. Antibody profiles may reflect immune recognition of proteins exposed during cellular stress or inflammation and may therefore represent potential biomarkers of disease-related alterations.

In this study, we investigated plasma antibody responses against a panel of viral and host targets in patients with Parkinson’s disease and healthy controls (HCs). Specifically, we analyzed antibodies against HSV-1, HERV-K, IRF5, CTSB, ASRGL1, interferon-α (IFN-α), interferon-ω (IFN-ω), α-synuclein, olfactory receptor proteins and DKK3. By comparing antibody levels and exploring their correlations, we aimed to identify immune patterns that may be associated with PD and to explore possible links between antiviral responses, interferon signaling and pathways related to protein homeostasis and cellular stress.

## Materials and methods

This study was performed in accordance with the principles of the Declaration of Helsinki. Written informed consent was obtained from all participants prior to their inclusion in the study. The study protocol was approved by the local Ethics Committee of the Azienda Ospedaliera-Universitaria of Sassari, Italy (IRB number 291123). All procedures involving human samples were carried out in accordance with applicable institutional and regulatory guidelines.

### Samples

Peripheral whole blood samples were obtained from individuals with a clinical diagnosis of PD and from healthy blood donors. A total of 50 PD patients were recruited between 2023 and 2025 (26 females and 24 males; median age = 75 years) at the Neurorehabilitation Service of the Azienda Ospedaliera-Universitaria. PD patients were stratified into subgroups according to disease severity based on the Hoehn and Yahr (HY) scale (HY 1-4, n = 42; HY 5, n = 8) with stage 5 representing the most advanced and severe stage of the disease, disease duration (1–10 years, n = 42; 11–20 years, n = 8), and sex (females, n = 26; males, n = 24). A cohort of 54 healthy control subjects was enrolled during 2024 at the Blood Transfusion Centre of Sassari (26 females and 28 males; median age = 55 years).

### Blood samples collection

Peripheral venous blood samples were collected from participants using K2-EDTA tubes. Whole blood was carefully layered in 15 mL tubes for density gradient separation in an equal volume of Ficoll (Sigma-Aldrich, St. Louis, MO, USA), followed by centrifugation at 1800 rpm for 20 minutes without brake. The plasma fraction obtained following centrifugation was collected and stored at -80 °C until analysis.

### Determination of antibodies by peptide ELISA

Epitope selection was performed using the Immune Epitope Database (IEDB - available at https://www.iedb.org - accessed August 2025), an online resource that integrates experimentally validated and computationally predicted immune epitopes. Candidate peptide sequences were selected based on predicted linear B-cell epitope characteristics, including antigenicity and surface accessibility, and were further refined to exclude sequence overlap with other known epitopic regions, thereby ensuring specificity of the antibody detection. Selected epitopes were synthesized at >95% purity (LifeTein, South Plainfield, NJ 07080, USA) and dissolved in dimethyl sulfoxide (DMSO) to obtain 10 mM stock solutions. The list of synthetic linear epitopes is reported in **Table 1**.

**Table 1:** List of epitope sequences used in the study.

Epitope	Sequence	Amino acid (aa) position
HERV-K	VWVPGPTDDRCPAKPEEEG	From aa 19 to aa 37
IRF-5	VVPVAARLLL	From aa 434 to aa 443
CTSB	EPGYSPTYKQDKHYGY	From aa 211 to aa 227
ASRGL1	PISKDRKERVHQ	From aa 14 to aa 25
IFN-α	GVGVTETPLMKEDSILA	From aa 103 to aa 119
IFN-ω	VGEGESAGAISSPALTLR	From aa 127 to aa 144
α-SYN	KNEEGAPQEGILEDMPVD	From aa 102 to aa 119
HSV-1	KNNYGSTIEGLLDLPDD	From aa 324 to aa 340
OLF-R	MSTRVCT	From aa 134 to aa 140
DKK3	PREVPDEYE	From aa 301 to aa 309

The table details the amino acid sequences of the epitopes used for antibody detection.

The indirect enzyme-linked immunosorbent assay (ELISA) was performed to detect antibody (Ab) reactivity against the selected peptides. Ninety-six-well microplates (Nunc) were coated overnight at 4 °C with 3 µM peptide diluted in 0.05 M carbonate-bicarbonate buffer (pH 9.5; Sigma). After coating, plates were blocked with 1% skimmed milk (Sigma) for 1 hour at 37 °C to prevent nonspecific binding and subsequently washed twice with Tris-buffered saline containing 0.05% Tween-20 (TBS-T). Plasma samples were diluted 1:20 in 1% skimmed milk prepared in TBS-T, and 100 µL of each diluted sample were added to the wells. Plates were incubated for 2 hours at 37 °C. Following incubation, plates were washed with TBS-T and incubated for 1 hour at 37 °C with an alkaline phosphatase-conjugated goat anti-human immunoglobulin G (IgG) polyclonal antibody (1:1000; Sigma). After four additional washes with TBS-T, plates were incubated with para-nitrophenyl phosphate substrate (Sigma) for 15 minutes at room temperature in the dark. Absorbance was measured at 405 nm using a microplate reader (Molecular Devices).

Each plasma sample was analyzed in triplicate, and the results represent the mean of two independent experiments performed under identical conditions. Blank values, wells coated with peptides and incubated with the secondary antibody alone, were subtracted from all sample readings. Positive and negative control plasma samples were included in each experiment. A reference plasma sample was included as a calibrator on each plate to minimize inter-plate variability. Results are expressed as the mean optical density (OD) values of triplicate measurements.

### Statistical analysis

ELISA optical density (OD) values were analyzed using non-parametric statistics due to their non-normal distribution. Differences in antibody reactivity between PD patients and healthy controls were assessed using the Mann-Whitney U test. Antibody positivity thresholds were determined by receiver operating characteristic (ROC) curve analysis, and group differences in seropositivity were subsequently evaluated using Fisher’s exact test.

Correlation analysis between antibody levels was performed using Spearman’s rank correlation coefficient (ρ) to evaluate potential associations among the analyzed targets. Correlation coefficients and corresponding p-values were calculated for each pairwise comparison. A p-value < 0.05 was considered statistically significant. Statistical analyses were performed using Graph Pad Prism 5.04.

## Results

**Figure 1:**
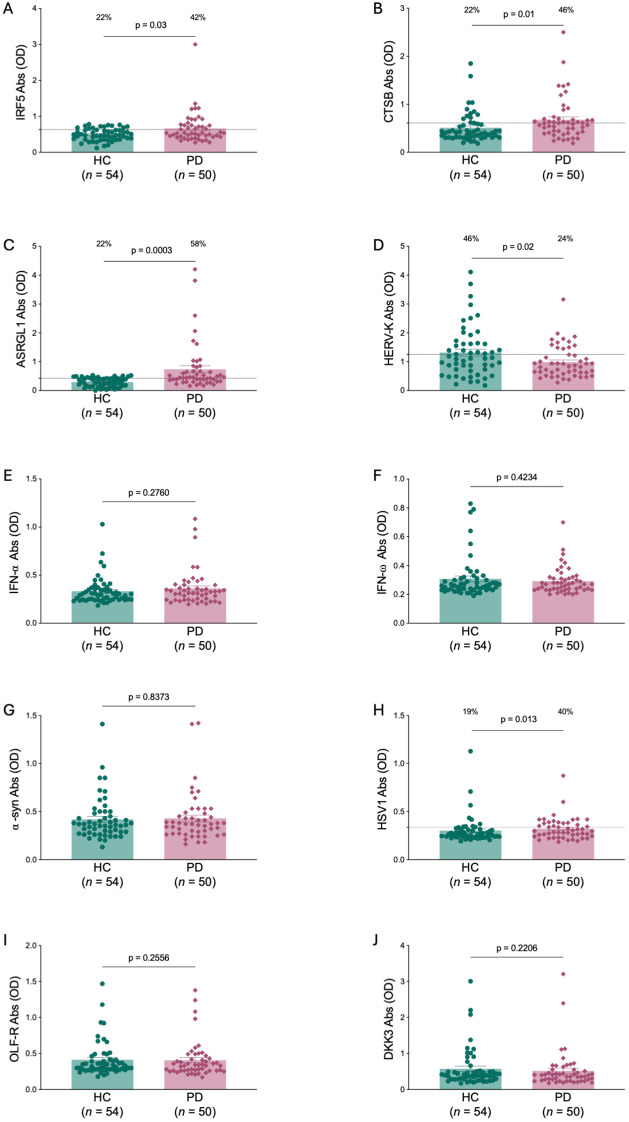
ELISA-based analysis of antibody reactivity against target epitopes. Plasma samples from patients with Parkinson and healthy controls (HC) were analyzed using an indirect ELISA for the detection of antibodies against IRF5 **(A)**, CTSB **(B)**, ASRGL1 **(C)**, HERV-K **(D)**, IFN-α **(E)**, IFN-ω **(F)**, α-syn **(G)**, HSV-1 **(H)**, OLF-R **(I)**, DKK3 **(J)**. Results are expressed as median ± standard error of the mean (SEM). Mann-Whitney p-values and the percentage of antibody-positive individuals, determined by Fisher’s exact test, are reported in the upper part of each graph. Positivity thresholds were established by receiver operating characteristic (ROC) curve analysis.

Plasma antibody levels against viral and host targets were compared between patients with Parkinson’s disease (PD; n = 50) and healthy controls (HC; n = 54). PD patients showed significantly higher antibody levels against IRF5 compared with controls (positivity: PD 42% vs HC 22%; Fisher’s exact test, p = 0.03) (**Figure 1A**). Similarly, antibody responses against CTSB were significantly increased in PD patients (positivity: PD 46% vs HC 22%; Fisher’s exact test, p = 0.01), (**Figure 1B**). Elevated antibody levels were also observed for ASRGL1 in the PD group compared with healthy controls (positivity: PD 58% vs HC 22%; Fisher’s exact test, p = 0.0003) (**Figure 1C**). In addition, antibodies against HSV-1 were significantly higher in PD patients than in controls (positivity: PD 40% vs HC 19%; Fisher’s exact test, p = 0.013) (**Figure 1H**). In contrast, antibody levels against HERV-K were significantly lower in PD patients compared with healthy controls (positivity: PD 24% vs HC 46%; Fisher’s exact test, p = 0.02) (**Figure 1D**).

No statistically significant differences between PD patients and controls were observed for antibodies against IFN-α (Mann-Whitney U test, p = 0.27), IFN-ω (Mann-Whitney U test, p = 0.42), α-syn (Mann-Whitney U test, p = 0.84), OLF-R (Mann-Whitney U test, p = 0.25) or DKK3 (Mann-Whitney U test, p = 0.22). (Figures E–G, I, J) It is important to highlight that after Benjamini-Hochberg FDR correction for multiple comparisons, ASRGL1 and CTSB remained statistically significant (q = 0.001 and q = 0.017, respectively), while IRF5 and HERV-K did not survive correction, and are therefore considered hypothesis-generating findings warranting validation in independent cohorts.

**Figure 2:**
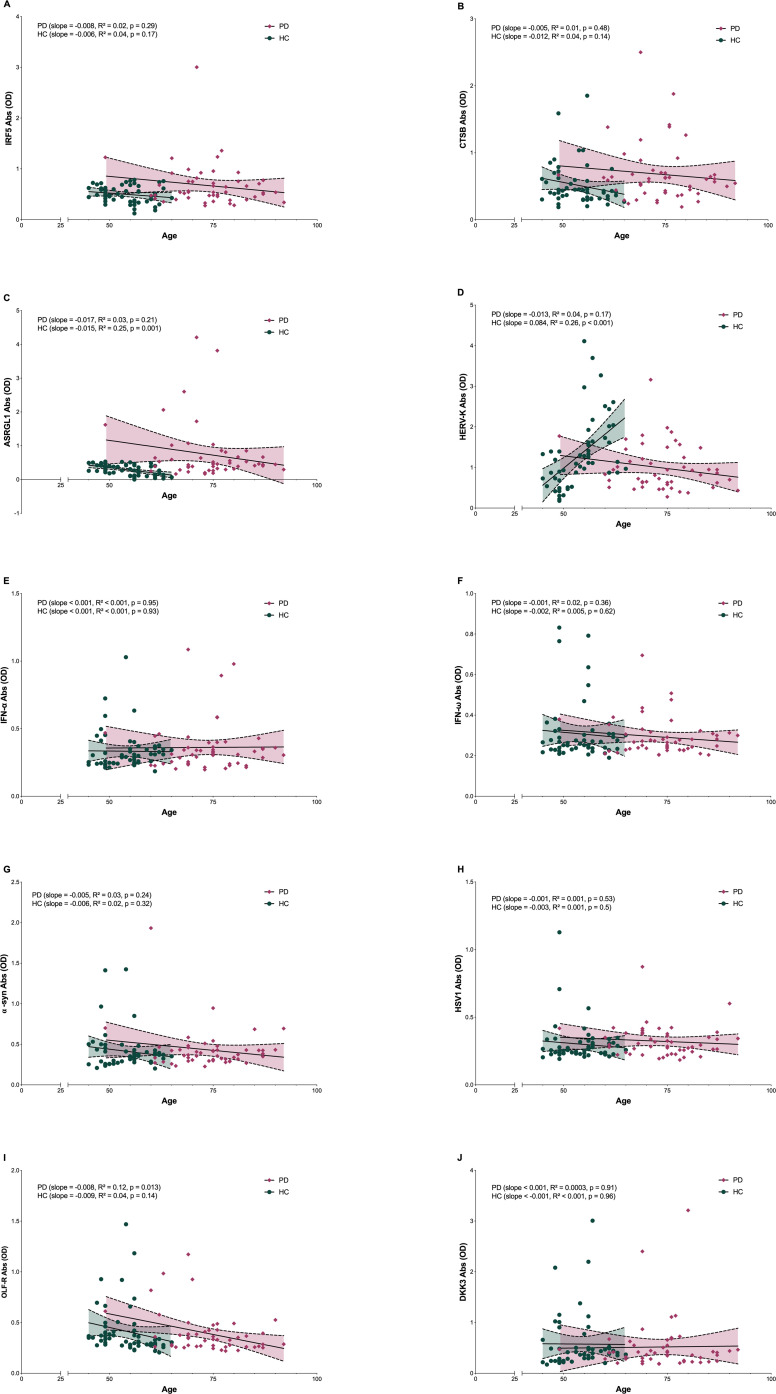
Simple linear regression of antibody levels versus age for each analyte, stratified by group. Scatter plots show antibody levels (OD values) against age (years) for PD patients and healthy controls for each target: IRF5 **(A)**, CTSB **(B)**, ASRGL1 **(C)**, HERV-K **(D)**, IFN-α **(E)**, IFN-ω **(F)**, α-syn **(G)**, HSV1 **(H)**, OLF-R **(I)**, and DKK3 **(J)**. Lines represent the best-fit linear regression for each group. The slope, R², and p-value for each regression are reported within each panel. A p-value < 0.05 was considered statistically significant.

Given the significant age difference between PD patients and HC in our cohort (median 75 vs 55 years), we performed multiple linear regression analyses to assess whether age could account for the observed differences in antibody levels. For each analyte, antibody levels (OD values) were used as the dependent variable, with age and group (PD = 1; HC = 0) as independent predictors (**Table 2**). The β coefficients for age were small and non-significant for the vast majority of targets (p > 0.05 for 9 out of 10 analytes), while group differences remained significant for the key targets of interest (IRF5, CTSB, ASRGL1, HERV-K), indicating that the group effect is independent of age. To further address the assumption of linearity underlying the regression model, we performed simple linear regression of antibody levels versus age separately for PD patients and HC for each analyte and added the resulting scatterplots as **Figure 2**. For IRF5 (A), no significant association with age was observed in either PD patients (slope = -0.008, R² = 0.02, p = 0.29) or HC (slope = -0.006, R² = 0.04, p = 0.17). For CTSB (B), no significant association with age was observed in either PD patients (slope = -0.005, R² = 0.01, p = 0.48) or HC (slope = -0.012, R² = 0.04, p = 0.14). For ASRGL1 (D), a significant negative association with age was observed in HC (slope = -0.015, R² = 0.25, p = 0.0001), but not in PD patients (slope = -0.017, R² = 0.03, p = 0.21). Since HC subjects were younger than PD patients in our cohort, and antibody levels in HC decrease with age, the higher antibody levels observed in PD patients in the primary analysis are unlikely to be driven by age differences between groups. If anything, age adjustment would be expected to further increase the observed PD-HC difference, strengthening our conclusions. For HERV-K (D), a significant positive association with age was observed in HC (slope = 0.084, R² = 0.26, p < 0.0001), but not in PD patients (slope = -0.013, R² = 0.04, p = 0.17). Notably, since HC subjects were younger than PD patients in our cohort, this age effect would be expected to reduce rather than inflate the observed difference between groups. Therefore, the significantly higher HERV-K antibody levels detected in HC compared to PD patients in the primary analysis are unlikely to be explained by age. For IFN-α (E), no significant association with age was observed in either PD patients (slope < 0.001, R² < 0.001, p = 0.95) or HC (slope < 0.001, R² < 0.001, p = 0.93). For IFN-ω (F), no significant association with age was observed in either PD patients (slope = -0.001, R² = 0.02, p = 0.36) or HC (slope = -0.002, R² = 0.005, p = 0.62). For alpha-synuclein (G), no significant association with age was observed in either PD patients (slope = -0.005, R² = 0.03, p = 0.24) or HC (slope = -0.006, R² = 0.02, p = 0.32). For HSV1 (H), no significant association with age was observed in either PD patients (slope = -0.001, R² = 0.001, p = 0.53) or HC (slope = -0.003, R² = 0.001, p = 0.50). For OLF-R (I), a modest negative association with age was observed in PD patients (slope = -0.008, R² = 0.12, p = 0.013), while no significant age effect was detected in HC (slope = -0.009, R² = 0.04, p = 0.14). However, since no significant difference between PD and HC was detected for this target in the primary analysis, this age association does not affect the main conclusions of the study. For DKK3 (J), the slope was not significantly different from zero in either PD (slope < 0.001, R² = 0.0003, p = 0.91) or HC (slope < -0.001, R² < 0.001, p = 0.96). Taken together, these results confirm the absence of a meaningful age-dependent trend in antibody levels, supporting the validity of our regression adjustment.

**Table 2:** Multiple linear regression analysis assessing the effect of age on antibody levels across all targets analyzed.

Epitope	β age	*p*	β group	*p*
IRF 5	-0.0071	0.198	0.3132	0.0045
CTSB	-0.0071	0.174	0.3078	0.017
ASRGL1	-0.0166	0.040	0.7779	0.0001
HERV-K	0.0152	0.135	-0.6342	0.011
IFN-alpha	4.312e-005	0.985	0.0272	0.626
IFN-omega	-0.0015	0.365	0.0144	0.726
Alpha-syn	-0.0024	0.476	0.0653	0.441
HSV1	-0.0025	0.161	0.0715	0.109
OLF-R	-0.0082	0.007	0.1414	0.056
DKK3	0.0005	0.945	-0.0671	0.711

For each target, multiple linear regression was performed using antibody levels (OD values) as the dependent variable and age and group (PD = 1; HC = 0) as independent predictors. The β coefficient represents the estimated change in antibody levels for each one-unit increase in the predictor variable (age in years, or group). A p value < 0.05 was considered statistically significant. Underlined targets are those that showed statistically significant differences in antibody levels between PD patients and healthy controls in the primary analyses.

**Figure 3:**
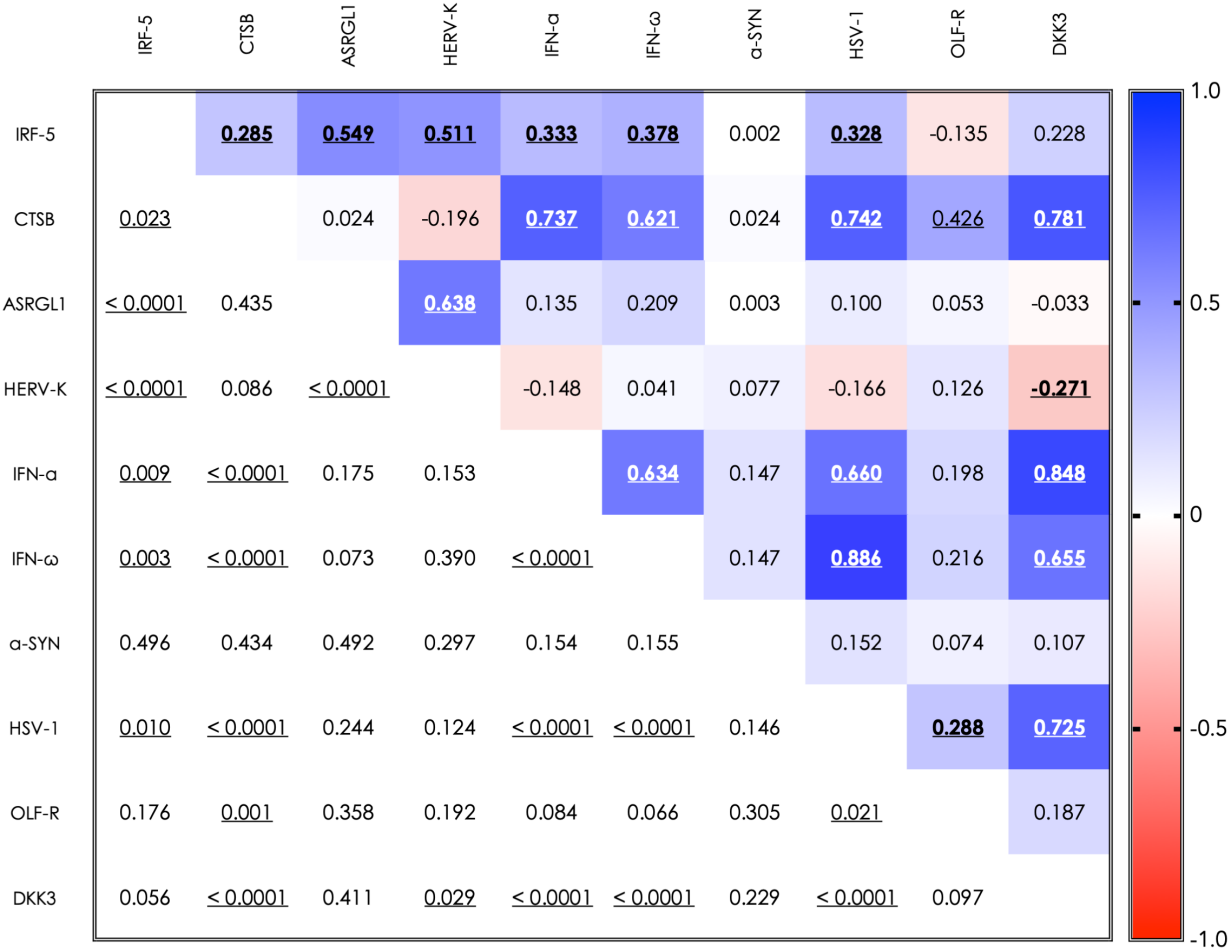
Correlation matrix of antibody levels against viral and host targets. Spearman’s rank correlation coefficients (ρ) are reported in the upper triangle (right side), while corresponding p-values are shown in the lower triangle (left side). Significant correlations are underlined. Color coding represents the direction and strength of the correlations, ranging from negative (red) to positive (blue). The bold labels indicate statistically significant R value.

Correlation analysis revealed several significant associations among antibody levels (**Figure 3**). Antibodies against IRF5 showed positive correlations with CTSB (Spearman’s r = 0.285, p = 0.023), ASRGL1 (Spearman’s r = 0.549, p < 0.0001), HERV-K (Spearman’s r = 0.511, p < 0.0001), IFN-α (Spearman’s r = 0.333, p = 0.009), IFN-ω (Spearman’s r = 0.378, p = 0.003) and HSV-1 (Spearman’s r = 0.328, p = 0.01). Antibodies against CTSB were positively correlated with IFN-α (Spearman’s r = 0.737, p < 0.0001), IFN-ω (Spearman’s r = 0.621, p < 0.0001), HSV-1 (Spearman’s r = 0.742, p < 0.0001), OLF-R (Spearman’s r = 0.426, p = 0.001) and DKK3 (Spearman’s r = 0.781, p < 0.0001).

ASRGL1 antibodies showed positive correlations with HERV-K (Spearman’s r = 0.638, p < 0.0001). IFN-α antibody levels correlated positively with IFN-ω (Spearman’s r = 0.634, p < 0.0001), HSV-1 (Spearman’s r = 0.66, p < 0.0001) and DKK3 (Spearman’s r = 0.848, p < 0.0001). Similarly, IFN-ω antibodies correlated with HSV-1 (Spearman’s r = 0.886, p < 0.0001) and DKK3 (Spearman’s r = 0.655, p < 0.0001). HSV-1 antibody levels also showed positive correlations with OLF-R (Spearman’s r = 0.288, p = 0.021) and DKK3 (Spearman’s r = 0.725, p < 0.0001).

Notably, a significant negative correlation was observed between HERV-K and DKK3 antibody levels (Spearman’s r = -0.271, p = 0.029). No additional significant correlations were identified for the remaining comparisons, as shown in **Figure 2**.

One of the main limitations of this study is the age difference between PD patients (median age: 75 years) and healthy controls (median age: 55 years). This represents a common challenge in this type of research, as recruiting elderly individuals without relevant comorbidities is inherently difficult. From a biological standpoint, aging is associated with significant immune remodeling, including immunosenescence and inflammaging, which may alter antibody production and overall humoral immune profiles. To assess whether the age difference between groups could represent a confounding factor, we performed multiple linear regression analyses including age as a covariate for all targets analyzed (**Table 2**). For most targets, age was not a significant predictor of antibody levels. Notably, for the differentially abundant targets IRF5, CTSB, and HERV-K, the group effect remained statistically significant after adjustment for age (*p* = 0.005, *p* = 0.017, and *p* = 0.011, respectively), while age itself was not a significant predictor, strongly supporting the conclusion that the observed differences reflect disease-associated immune responses rather than age-related changes. For ASRGL1, both group (*p* = 0.0001) and age (*p* = 0.040) were significant predictors. However, the strong and highly significant group effect suggests that the differential antibody response is primarily driven by disease status rather than aging alone. For the remaining targets, neither age nor group reached statistical significance, consistent with the absence of differential antibody responses observed in the primary analyses.

**Figure 4:**
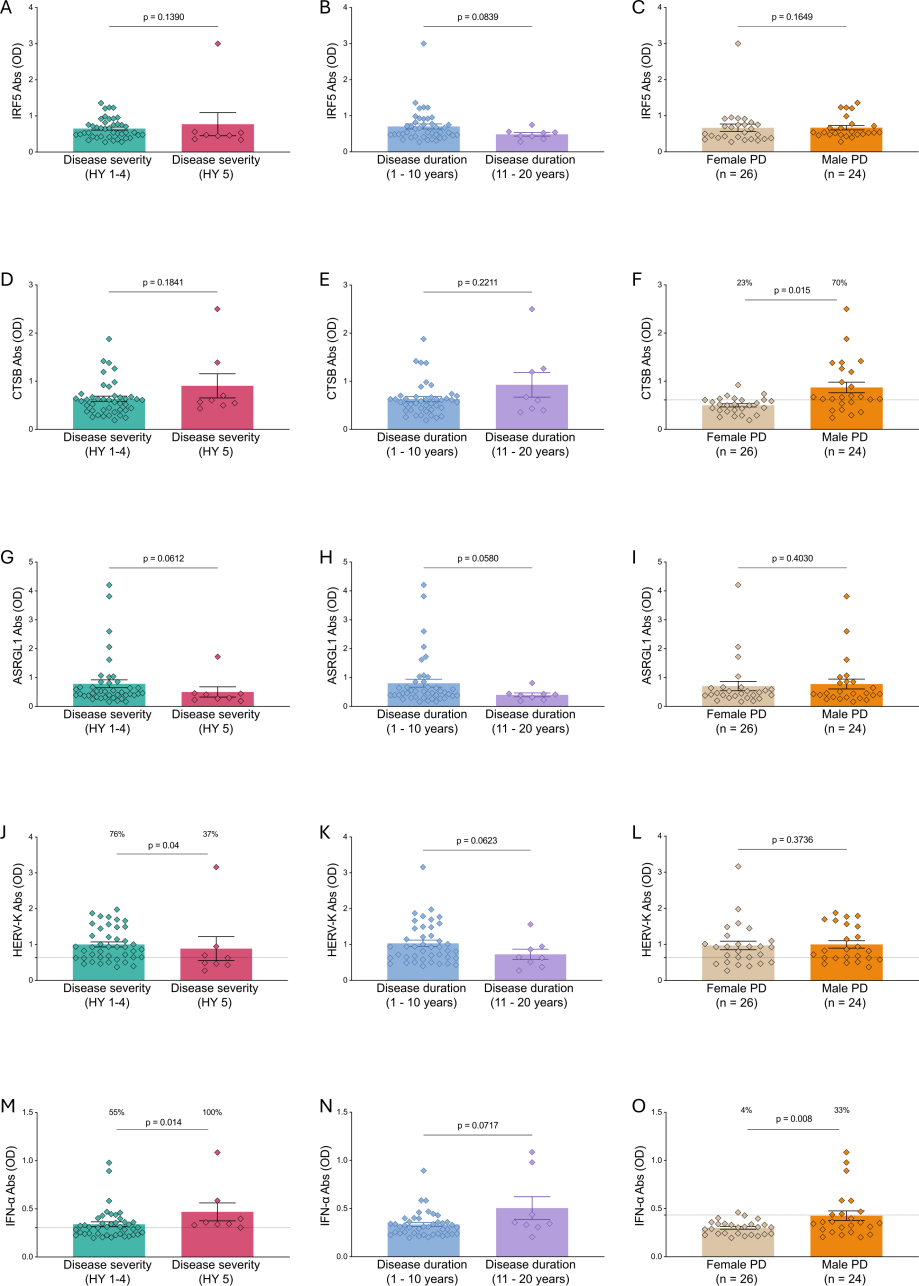
ELISA-based analysis of antibody reactivity against target epitopes. Plasma samples from patients with Parkinson’s disease were stratified into subgroups according to disease severity (HY 1-4, n = 42; HY 5, n = 8), disease duration (1–10 years, n = 42; 11–20 years, n = 8), and sex (females, n = 26; males, n = 24), and analyzed by indirect ELISA to detect antibodies against IRF5 **(A–C)**, CTSB **(D–F)**, ASRGL1 **(G–I)**, HERV-K **(J–L)**, IFN-α **(M–O)**. Results are expressed as median ± standard error of the mean (SEM). Mann-Whitney U test p-values and the percentage of antibody-positive individuals, determined by Fisher’s exact test, are reported in the upper part of each graph. Positivity thresholds were defined based on receiver operating characteristic (ROC) curve analysis.

**Figure 5:**
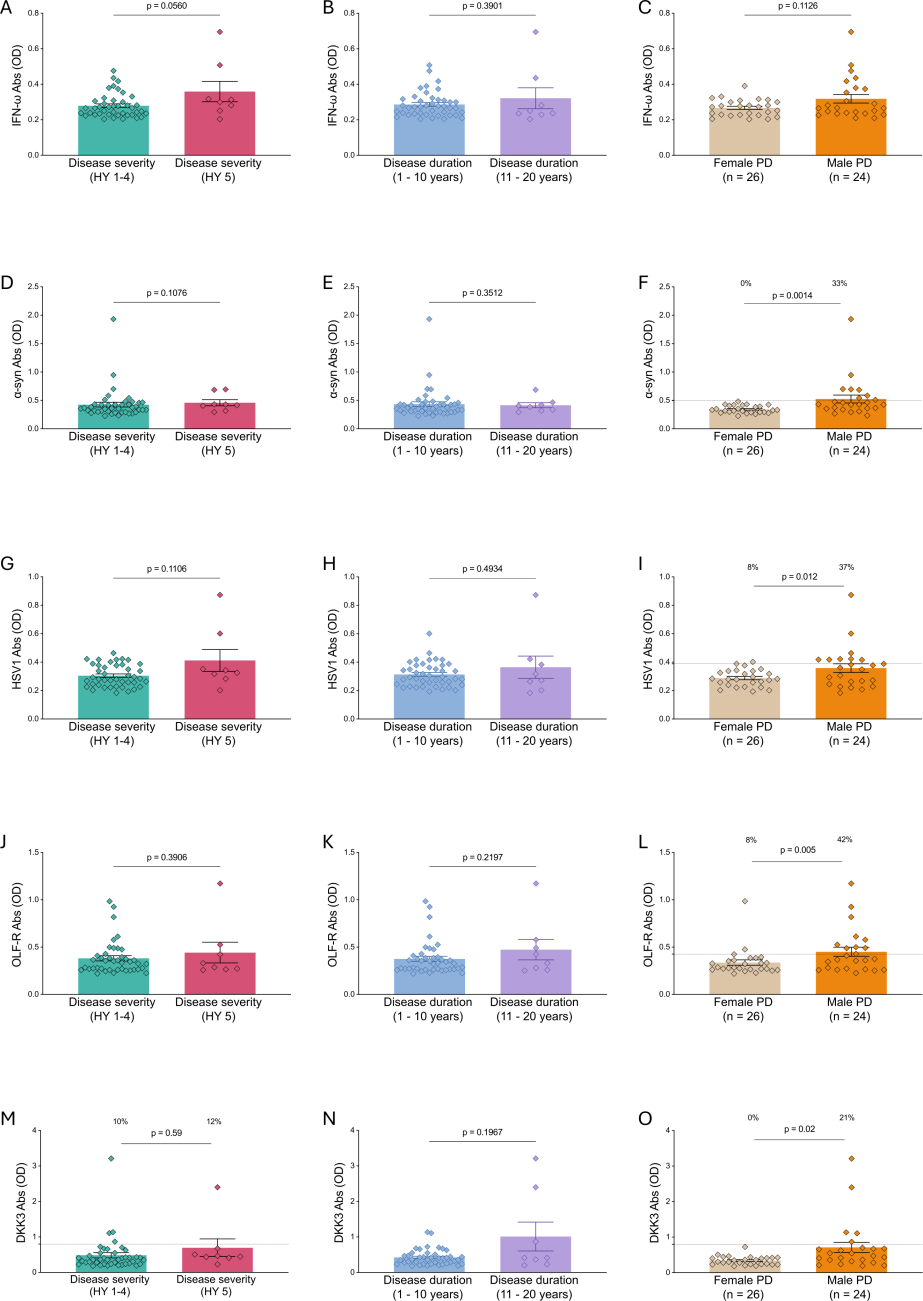
ELISA-based analysis of antibody reactivity against target epitopes. Plasma samples from patients with Parkinson’s disease were stratified into subgroups according to disease severity (HY 1-4, n = 42; HY 5, n = 8), disease duration (1–10 years, n = 42; 11–20 years, n = 8), and sex (females, n = 26; males, n = 24), and analyzed by indirect ELISA to detect antibodies against IFN-ω **(A–C)**, α-synuclein **(D–F)**, HSV-1 **(G–I)**, OLF-R **(J–L)**, and DKK3 **(M–O)**. Results are expressed as median ± standard error of the mean (SEM). Mann-Whitney U test p-values and the percentage of antibody-positive individuals, determined by Fisher’s exact test, are reported in the upper part of each graph. Positivity thresholds were defined based on receiver operating characteristic (ROC) curve analysis.

To further explore potential associations with clinical variables, subgroup analyses were performed by stratifying PD patients according to disease severity and sex. Differences were mainly observed in relation to Hoehn and Yahr (HY) stage and patient sex (**Figures 4**, **5**). In particular, higher antibody levels against HERV-K were detected in patients with HY stages 1–4 compared with those classified as HY stage 5 (positivity: HY_1-4_ 76% vs HY5 19%; Fisher’s exact test, p = 0.04) (**Figure 4J**). In contrast, antibody levels against IFN-α were higher in patients with HY stage 5 compared with those in stages 1-4 (positivity: HY_1-4_ 55% vs HY5 100%; Fisher’s exact test, p = 0.014), (**Figure 4M**).

Sex-related differences were also observed in the humoral response. Female patients showed lower antibody levels compared with male patients for several targets, including CTSB (positivity: F 23% vs M 70%; Fisher’s exact test, p = 0.015), (**Figure 4F**), IFN-α (positivity: F 4% vs M 33%; Fisher’s exact test, p = 0.008), (**Figure 4O**), α-synuclein (positivity: F 0% vs M 33%; Fisher’s exact test, p = 0.0014) (**Figure 5F**), HSV-1 (positivity: F 8% vs M 37%; Fisher’s exact test, p = 0.012) (**Figure 5I**), OLF-R (positivity: F 8% vs M 42%; Fisher’s exact test, p = 0.005), (**Figure 5L**) and DKK3 (positivity: F 0% vs M 21%; Fisher’s exact test, p = 0.02). (**Figure 5O**) No significant differences were observed when patients were stratified according to disease duration. (**Figures 4**, **5**).

## Discussion

In this study, we examined antibody responses against a panel of viral and host proteins in patients with Parkinson’s disease and HCs. Among the analyzed targets, HERV-K emerged as the most distinctive potential marker, showing significantly lower antibody levels in PD patients compared with controls. In contrast, increased antibody responses were observed against IRF5, CTSB, ASRGL1, and HSV-1, while no differences were detected for IFN-α, IFN-ω, α-synuclein, OLF-R, and DKK3. Overall, these findings suggest that immune alterations in PD are selective and involve specific pathways rather than reflecting a generalized increase in humoral activity.

The reduced antibody response against HERV-K represents a key observation of this study. Human endogenous retroviruses are integrated elements (Simula et al., 2025) of the human genome whose expression can be modulated by inflammatory (Rangel et al., 2022) and interferon-related signals (Wang et al., 2022). The lower levels of anti-HERV-K antibodies observed in PD patients suggest that immune recognition of this retroelement may be altered in the disease. Interestingly, despite this overall reduction, HERV-K remained connected to other components of the immune response, showing positive correlations with IRF5 and ASRGL1, and a negative correlation with DKK3 suggesting that HERV-K-related immune responses may be linked both to interferon-dependent pathways and to mechanisms involved in cellular homeostasis. A particularly relevant aspect is that HERVs are generally recognized as targets of the humoral response in neurodegenerative, autoimmune and other conditions, which already introduces a distinguishing element in the context of PD, where HERV-K may represent a specific and differential target (Arru et al., 2020; Noli et al., 2022; Simula et al., 2023; Bo et al., 2024; Fayyaz et al., 2025).

The association between HERV-K and IRF5 is particularly noteworthy. IRF5 is a key regulator of innate immune responses and plays a central role in type I interferon signaling (Wang et al., 2024). In our results, antibodies against IRF5 were increased in PD patients and showed multiple correlations with other targets, including HERV-K, CTSB, ASRGL1, IFN-α, IFN-ω, and HSV-1. Although antibody levels against interferons themselves did not differ between PD subgroups, these associations suggest that interferon-related immune responses may be involved in the broader immune context observed in PD. The link between HERV-K and IRF5 may therefore reflect a connection between endogenous retroviral elements and interferon-driven immune pathways.

HERV-K also showed a positive association with ASRGL1, a protein involved in amino acid metabolism and protein homeostasis (Cantor et al., 2009). While the role of ASRGL1 in PD is not well defined, its correlation with HERV-K suggests that changes in immune recognition of endogenous retroelements may occur in parallel with alterations in metabolic and proteostatic processes. This is further supported by the increased antibody levels observed against CTSB, a lysosomal protease involved in protein degradation (Wang et al., 2023). CTSB antibodies were elevated in PD patients and correlated with several immune-related markers, including interferons and HSV-1, indicating that lysosomal and degradation pathways may be linked to the immune alterations detected in this study. We recently showed that Cathepsin B may exert a protective role in the context of Parkinson’s disease, as genetically predicted higher levels have been associated with a reduced risk of disease onset (Lu et al., 2024). In this perspective, the increased antibody response against CTSB observed in our study may be of particular relevance, as the presence of autoantibodies could potentially interfere with its physiological function.

The negative correlation observed between HERV-K and DKK3 provides an additional layer of interpretation. DKK3 is a modulator of the Wnt signaling pathway (Nakamura and Hackam, 2010) and has been associated with cellular stress responses (Song et al., 2024) and tissue remodeling (Zhou et al., 2025). The inverse relationship between HERV-K and DKK3 may suggest that reduced immune recognition of HERV-K is associated with increased activation of pathways related to cellular stress, although this hypothesis requires further investigation.

The increased antibody levels observed against HSV-1 further indicate the involvement of antiviral immune responses in PD patients. HSV-1 is a neurotropic virus capable of establishing latency and undergoing periodic reactivation (Nicoll et al., 2012), which can stimulate interferon signaling (Whitford et al., 2025). In our study, HSV-1 antibodies correlated with IRF5 and interferon-related markers, suggesting that antiviral responses and interferon pathways may be interconnected. However, the lack of a direct correlation between HSV-1 and HERV-K suggests that these two components may reflect distinct aspects of the immune response in PD, with HSV-1 representing exogenous antiviral stimulation and HERV-K reflecting endogenous regulatory processes. In this context, it is important to note that HSV-1 has been described as a transactivator of HERVs (Perron et al., 1993). However, in the present study we are evaluating the humoral response, which does not directly capture this aspect. It would therefore be of particular interest to assess gene expression levels of both targets in the same patients to determine whether a positive correlation exists at the transcriptional level. In our previous work, we showed that infection of neuronal SH-SY5Y cells can lead to HERV-K reactivation (Jasemi et al., 2025). However, this relationship may not necessarily be reflected at the level of the humoral response, as observed in the present study. This aspect may be linked to active HSV-1 infection, which cannot be directly assessed by our assay. Nevertheless, prior exposure to HSV-1 appears to be common in the majority of PD patients.

In the present study, no significant differences in antibody levels against α-synuclein were observed between PD patients and HCs. This finding is consistent with the existing literature, as several studies have reported comparable anti-α-synuclein autoantibody levels between PD patients and controls, and a systematic review and meta-analysis has concluded that α-synuclein autoantibodies lack sufficient consistency to serve as reliable biomarkers for PD (Heinzel et al., 2014; Scott et al., 2018). From a biological standpoint, α-synuclein is predominantly an intracellular protein, and its limited extracellular exposure under physiological conditions may restrict its accessibility to the humoral immune system, consequently limiting the generation of a robust and disease-specific antibody response. Furthermore, the aggregated and modified forms of α-synuclein that accumulate in PD may not be efficiently presented to B cells in an immunogenic context sufficient to elicit a differential humoral response. These suggest that the humoral immune response against α-synuclein does not discriminate between PD patients and healthy individuals, further supporting that anti-α-synuclein antibodies do not represent a suitable serological biomarker for PD.

Stratification analyses provided additional insight into the behavior of HERV-K in relation to clinical features. Patients with earlier disease stages (HY 1-4) showed higher antibody levels against HERV-K compared with those with advanced disease (HY5), suggesting that the reduction in HERV-K-related immune responses may become more pronounced with disease progression. This pattern appears to differ from what has been reported in other neurodegenerative conditions, such as amyotrophic lateral sclerosis (ALS), where antibody responses against HERV-K have been shown to increase with disease progression and have been proposed to exert a potential protective effect in more advanced stages (Garcia-Montojo et al., 2022).

In contrast, antibodies against IFN-α were higher in patients with HY5, supporting the idea that interferon-related responses may increase in more advanced stages. Although exploratory, these suggest that different components of the immune response may change over the course of the disease and could potentially reflect disease progression, with a possible predictive or indicative value for patient status. The observation that anti-HERV-K antibody levels are lower in PD patients compared to healthy controls and further reduced in advanced disease stages (HY5), argues against a straightforward model of HERV-K reactivation driving the immune response. Instead, this progressive decline may reflect a state of immune tolerance or humoral immune exhaustion toward HERV-K antigens, potentially developing as the disease advances. Alternatively, the reduction could indicate a broader impairment of B-cell-mediated immunity in late-stage PD, consistent with the immunosenescence observed in advanced neurodegeneration. In contrast, the increase in anti-IRF5 antibodies with disease progression suggests that interferon regulatory pathways become increasingly activated as the disease advances.

Sex-related differences were also observed, with female patients generally showing lower antibody levels than males for several targets, including CTSB, IFN-α, α-synuclein, HSV-1, OLF-R, and DKK3. These findings are consistent with known differences in immune responses between males and females (Bovenzi et al., 2025; Cattaneo and Pagonabarraga, 2025) and may contribute to variability in antibody profiles observed in the study population. In contrast, no differences were observed when patients were stratified according to disease duration, suggesting that the immune alterations detected in this study may not be directly related to disease length.

One of the main limitations of this study is the age difference between PD patients (median age: 75 years) and healthy controls (median age: 55 years). Multiple linear regression analyses including age as a covariate demonstrated that, for the differentially abundant targets, the group effect remained statistically significant independently of age (**Table 2**), suggesting that the observed findings are unlikely to be solely driven by age-related immune differences. To further verify the absence of age-dependent trends, we performed simple linear regression of antibody levels versus age for each analyte separately in PD and HC. For the majority of the targets, the slope was not significantly different from zero in either group, confirming the absence of a meaningful age-dependent effect. Where a significant age association was observed in HC (ASRGL1 and HERV-K), the direction of the effect would be expected to reduce rather than inflate the observed PD-HC differences, further supporting the validity of our conclusions. Nevertheless, given the potential relevance of this variable, further studies with age-matched cohorts are warranted to fully elucidate the contribution of age to the observed antibody profiles.

Additionally, we acknowledge that several established and emerging biomarkers for Parkinson’s disease have been reported in recent literature. However, the present study was designed using a hypothesis-driven approach, focusing on a selected panel of genes involved in interferon signaling, innate immune response, lysosomal pathways and protein clearance pathway, and endogenous retroviral activity. As such, other relevant biomarkers, such as α-synuclein species, neurofilament light chain, and additional inflammatory or omics-derived markers, were not included in the current analysis. Furthermore, the study does not determine whether the observed antibody responses play a direct functional role in disease pathogenesis or represent a secondary epiphenomenon associated with neurodegeneration, like α-synuclein accumulation. Furthermore, the functional role of the detected antibodies remains to be elucidated, as data on IgG subclass, complement activation, and immune complex formation were not assessed in this study. Future investigations addressing these aspects will be essential to determine whether the identified antibodies actively contribute to disease pathology or represent epiphenomena of immune dysregulation associated with Parkinson’s disease. These represent limitations of the study, and future investigations integrating broader biomarker panels will be necessary to further validate and extend these findings. In addition, we plan to validate these findings by assessing both gene expression and protein levels of the analyzed targets, and to perform functional studies to further elucidate their potential role in disease mechanisms, which will strengthen the overall impact of the study. The overall aim of this work is to identify novel potential biomarkers that can be easily detected across different stages and clinical contexts of the disease.

## Conclusions

Our results indicate that PD patients show selective changes in antibody responses involving antiviral pathways, interferon signaling, and proteins associated with cellular homeostasis. Within this context, HERV-K stands out as a distinctive potential marker, characterized by reduced antibody levels and specific associations with interferon-related and proteostasis-related targets. Although the biological mechanisms underlying these findings remain to be clarified, the targets identified in this study may represent potential biomarkers of immune alterations associated with Parkinson’s disease. Further studies will be necessary to determine whether these antibody responses have a functional role or primarily reflect immune changes associated with disease progression.

## Data Availability

The raw data supporting the conclusions of this article will be made available by the authors, without undue reservation.
